# Laparoscopic and endoscopic cooperative surgery for intra-mucosal gastric carcinoma adjacent to the ulcer scars

**DOI:** 10.1186/s12957-018-1355-0

**Published:** 2018-03-12

**Authors:** Masahiko Aoki, Satoshi Tokioka, Ken Narabayashi, Akitoshi Hakoda, Yosuke Inoue, Naoki Yorifuji, Yoshihide Chino, Isao Sato, Yutaro Egashira, Toshihisa Takeuchi, Kazuhide Higuchi

**Affiliations:** 1 Internal Medicine of Gastroenterology, First Towakai Hospital, Takatsuki, Osaka 569-0081 Japan; 2 Internal Medicine (II), Osaka Medical College, Takatsuki, Osaka 569-8686 Japan; 3 Endoscopic Surgery Center, First Towakai Hospital, Takatsuki, Osaka 569-0081 Japan; 4 Department of Pathology, Osaka Medical College, Takatsuki, Osaka 569-8686 Japan

**Keywords:** Laparoscopic and endoscopic cooperative surgery (LECS), Gastric carcinoma, Ulcer scar, Endoscopic submucosal dissection (ESD)

## Abstract

**Background:**

Laparoscopic and endoscopic cooperative surgery (LECS) was performed for the local resection of gastrointestinal stromal tumors (GIST). LECS enables less resection of the lesion area and preserves function. Furthermore, LECS can be safely performed and independent of tumor location. However, LECS is not usually used for cases involving gastric carcinoma because it may seed tumor cells into the peritoneal cavity when the gastric wall is perforated. Here, we report seven cases of LECS for intra-mucosal gastric carcinoma, which were difficult to carry out by endoscopic submucosal dissection (ESD) because of ulcer scars.

**Methods:**

We performed LECS (classical LECS and inverted LECS) in seven cases of intra-mucosal gastric carcinoma. All cases had ulcer scars beside the tumor. LECS was chosen because ESD was thought to be difficult because of the ulcer scars. We only selected cases in which the patients did not prefer gastrectomy and endoscopic examination was indicative of intra-mucosal gastric carcinoma.

**Results:**

In all cases, LECS was performed without severe complications including postoperative stenosis. Histopathology findings proved that the tumors were intra-mucosal carcinoma and had been resected completely. Furthermore, there were ulcer scars (Ul IIIs-IVs) beside the tumor. Currently, dissemination and recurrence have not been apparent.

**Conclusions:**

LECS for intra-mucosal gastric carcinoma is an efficient procedure, but strict observation is necessary because of the possibility of peritoneal dissemination. Results suggest that LECS is likely to be effective for cases involving intra-mucosal gastric carcinoma that are difficult to treat by ESD due to ulcer scars.

## Background

Laparoscopic and endoscopic cooperative surgery (LECS) is routinely performed for the local resection of gastrointestinal stromal tumors (GIST) [1–4]. This procedure has been on the national insurance list since February 2014 in Japan. LECS is an endoscopic dissection of the mucosal to submucosal layers followed by laparoscopic seromuscular resection and is independent of tumor location. Incision lines are determined, and a mucosal to submucosal incision is performed endoscopically, while the seromuscular layer is incised and the incision line is laparoscopically closed.

However, LECS is not usually used in cases involving gastric carcinoma because it may seed tumor cells into the peritoneal cavity if the gastric wall is perforated.

In general, endoscopic submucosal dissection (ESD) is applied for early gastric cancer [5, 6]. Endoscopic resection is less invasive than conventional surgery [7]. In our cases, we performed LECS for early gastric carcinoma because ulcer scars were located close by; consequently, ESD was likely to be very difficult to perform, leading to an expectation of a more complicated procedure and a broader range of dissection owing to the scarring. Furthermore, reports have shown that the larger the lesion, the higher the incidence of bleeding and perforation [8]. Also, while stricture is known to occur after ESD [9, 10], LECS results in no postoperative transformation of the remaining stomach [1]. We choose LECS to prevent these complications, and only selected cases involving patients who did not prefer gastrectomy and in which endoscopic examination was indicative of intra-mucosal gastric carcinoma. We performed LECS after fully explaining the procedure to the patients and with the permission of the local ethics committee. In the beginning, we performed classical LECS in four cases. Peritoneal dissemination has not yet been reported in these cases. However, to prevent the seeding of tumor cells into the peritoneal cavity when the gastric wall was perforated, we have performed inverted LECS [11] since February 2016.

## Methods

We retrospectively assessed seven cases at the First Towakai Hospital. Patients were 66- to 91-year-old male. We choose the cases where endoscopic examination was indicative of intra-mucosal gastric carcinoma adjacent to the ulcer scars, so ESD was thought to be difficult. Also, the patients preferred to avoid a gastrectomy. All procedures followed were in accordance with the ethical standards of the responsible committee on human experimentation (institutional and national), with the Declaration of Helsinki of 1964 and later versions, and also with the permission of the First Towakai Hospital IRB/ethics committee. Informed consent or a substitute for it was obtained from all patients for their being included in the study. Consent, for the publication of the case reports and any additional related information, was taken from the patients involved in the study. From January 2014 to February 2017, we performed classical LECS in four cases and inverted LECS in three cases of intra-mucosal gastric carcinoma. The surgical procedure first involved processing the serosal side laparoscopically around the tumor. We then used endoscopy to determine the incision line and entire circumference of the mucosal incision using an IT-2 knife. We used a needle knife to perforate the gastric wall and then used the IT-2 knife to make a full-thickness incision. After the incision using the IT-2 knife, we inserted an ultrasonically activated device into the perforation hole and accomplished the incision. A specimen was taken out from the mouth side or naval incision. The incision line was then closed using a laparoscopic hand-suturing technique. When a specimen was taken from the ventral side, we quickly recovered it and placed it into a specimen bag as soon as possible.

**Fig. 1:**
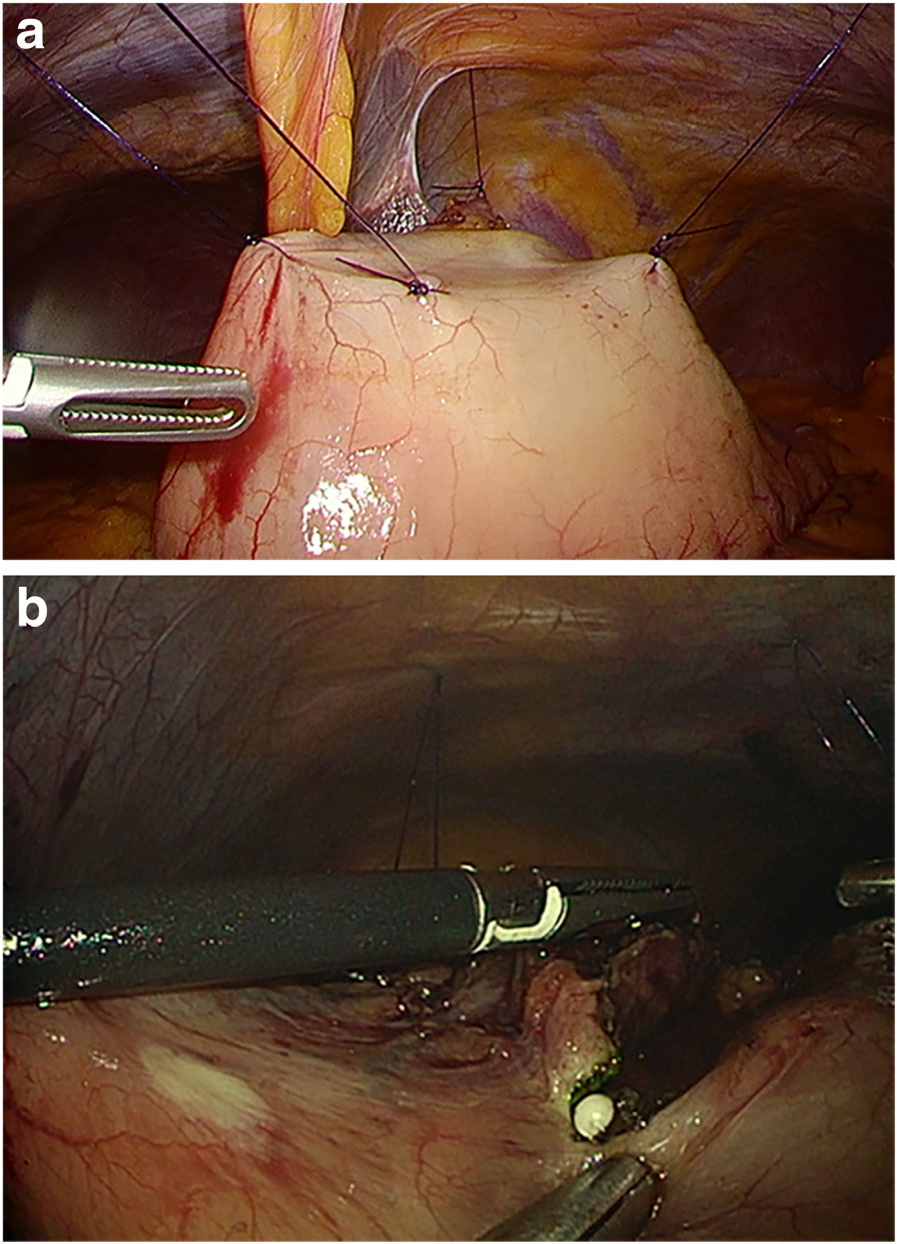
**a** The gastric wall was lifted up circumferentially outside of the incision line by several stitches. **b** The tip of the IT-2 knife was inserted into the perforation and a full-thickness incision was carried out under laparoscopy

In the case of inverted LECS, after determining the incision line endoscopically, the gastric wall was lifted up circumferentially outside the incision line by several stitches resembling a crown (Fig. 1). This procedure made the tumor turn towards the intra-gastric cavity when the gastric wall was perforated.

## Results

Mean patient age for the classical and inverted LECS cases was 78.8 ± 9.3 years and 79.0 ± 3.6 years, respectively. Mean operation time was 181.5 ± 37.9 min and 192.3 ± 51.9 min, for the two types of surgery, with a mean blood loss of 11.3 ± 5.4 ml and 11.0 ± 6.5 ml, for classical and inverted LECS, respectively. Mean length of postoperative hospital stay was 16.3 ± 2.1 days and 17 ± 5.1 days, respectively (Table 1). In all cases, there were no postoperative complications including stenosis. At the time of writing, dissemination and recurrence have not been recognized during follow-up (Table 2). Histopathology findings proved that tumors were intra-mucosal carcinomas and had been resected completely. Furthermore, there were ulcer scars (Ul IIIs-IVs) beside the tumors. In here, we listed one representative case of each LECS procedure.

**Table 1:** Characteristics and operative data for cases involving laparoscopic and endoscopic cooperative surgery (LECS)

		Classical (*n* = 4)	Inverted (*n* = 3)
Sex (male/female)		4/0	3/0
Age (years)		78.8 ± 9.3	79.0 ± 3.6
Location of tumor			
	Angle, lesser curvature	2	2
	Body, posterior	1	
	Body, lesser curvature	1	
	Body, greater curvature		1
Tumor size (mm)		14.5 ± 3.6 (10–20)	11.7 ± 6.2 (5–20)
Operation time (min)		181.5 ± 37.9	192.3 ± 51.9
Intraoperative blood loss (ml)		11.3 ± 5.4	11.0 ± 6.5
Conversion to open surgery		0	0
Postoperative complications		0	0
Gastric fullness		0	0
Anastomotic leakage		0	0
Anastomotic stenosis		0	0
Anastomotic bleeding		0	0
Postoperative hospital stay (days)		16.3 ± 2.1	17.0 ± 5.1

**Table 2:** Follow-up and passage after laparoscopic and endoscopic cooperative surgery (LECS)

Case	Age (years)	Sex	Tumor size (mm)	Classical/inverted	Follow-up after LECS (image)	Passage after LECS
1	91	Male	20	Classical	Endoscopy; 3, 9, 20 months CT; 3 months	No dissemination and recurrence, 42 months after LECS (alive)
2	66	Male	10	Classical	Endoscopy; 6, 18, 20 months CT; 16 months	No dissemination and recurrence, 35 months after LECS (alive)
3	75	Male	13	Classical	Endoscopy; 3 months CT; 1, 21 months	No dissemination and recurrence, died of pneumonia 24 months after LECS
4	83	Male	15	Classical	Endoscopy; 15, 27 months CT; 11, 17, 23, 29 months	No dissemination and recurrence, 33 months after LECS (alive)
5	82	Male	20	Inverted	Endoscopy; 3, 6, 11 months CT; 7, 12 months	No dissemination and recurrence, 18 months after LECS (alive)
6	74	Male	10	Inverted	Endoscopy; 3 months CT; 2, 4, 7 months	No dissemination and recurrence, 14 months after LECS (alive)
7	81	Male	5	Inverted	Endoscopy; 3 months	No dissemination and recurrence, 6 months after LECS (alive)

*LECS* laparoscopic and endoscopic cooperative surgery, *CT* computed tomography

**Fig. 2:**
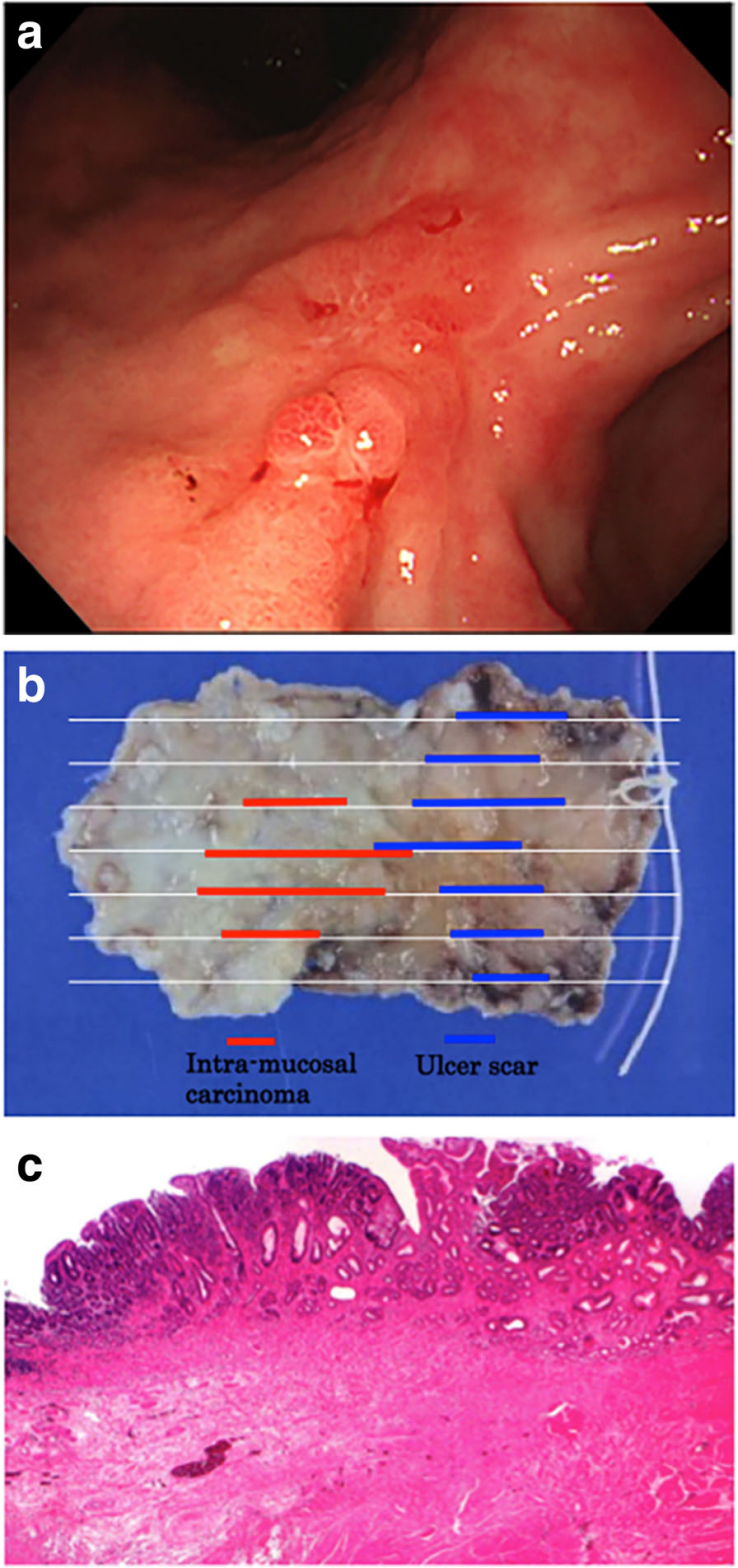
Endoscopic image (**a**) showing an intra-mucosal carcinoma of an ulcer scar, along with a pathological specimen (**b**) and histopathological image (**c**) of case 1

Case 1 is a 91-year-old male. Tumor size was 20 mm and located at the angle of the lesser curvature. Ulcer scar was on the anal side of the tumor. Classical LECS was performed without complication. Histopathology findings proved that the tumor was intra-mucosal carcinoma and was resected completely. Ulcer scars (Ul IIIs-IVs) were evident beside the tumor (Fig. 2).

Case 2 is an 82-year-old male. Tumor size was 20 mm and located at the angle of the lesser curvature. Ulcer scar was located on the posterior side of the tumor. Inverted LECS was performed without complication. Histopathology findings proved that the tumor was intra-mucosal carcinoma and was resected completely. Ulcer scars (Ul IIIs-IVs) were evident beside the tumor.

## Discussion

Intra-mucosal gastric cancer carries a low risk of lymph node metastasis [12]. ESD is indicated as a standard treatment (absolute indication or expanded indication) [13]. In our cases, the tumors were predicted as intra-mucosal gastric carcinomas, as a result of endoscopic examination. However, it was expected that the procedure would be complicated if ESD was performed. The greater the degree of submucosal fibrosis, the longer an ESD procedure can last and the higher the frequency of complications such as perforation and immediate bleeding [14]. In order to carry out the dissection safely with an accurately cut line, and to avoid excessive resection of the gastric wall, we choose to use LECS for our cases. LECS was successfully applied in cases of intra-mucosal gastric carcinomas which would have been difficult to treat with ESD due to ulcer scars. In all cases, there was no postoperative stricture, which may occur if ESD was performed. In a previous study, LECS was also performed for lateral-spreading mucosal gastric cancer which would have been difficult to treat with ESD because of the high incidence of complications and the long surgical time required for ESD [11].

Previous reports have shown that gastric perforation during endoscopic resection for gastric carcinoma does not lead to peritoneal dissemination, even in the long term [15]. However, we need to try not to seed tumor cells into the peritoneal cavity to prevent peritoneal dissemination when the gastric wall is perforated during the LECS procedure. Thus, in the inverted LECS procedure, the gastric wall is lifted up circumferentially outside of the incision line by several stitches [11]. This procedure enables us to prevent contact between the tumor and visceral tissue and prevents gastric juice from falling into the peritoneal cavity. Other techniques such as “CLEAN-NET” or “NEWS” [16–18] also prevent peritoneal dissemination by allowing non-exposure gastric wall resection. However, the mucosal layer shifts significantly from the seromuscular layer during surgery, so that they are not applied for tumor located at the EGJ or pyloric ring, and also the muscle layer and seromuscular layer may be incorrectly dissected. On the other hand, LECS can be applied for any tumor location [19]. Thus, LECS is likely to be effective in these cases, although strict observation is necessary for LECS in cases of intra-mucosal gastric carcinoma.

Due to ulcer scars located in the vicinity of a tumor, sometimes it is difficult to diagnose whether the tumor represents intra-mucosal carcinoma or submucosal infiltration carcinoma. On the other hand, sentinel node mapping for early gastric cancer has been reported [20, 21]; detection rate and the accuracy of prediction of lymph node metastasis based on sentinel status are of high value. In the future, combining sentinel node mapping technology and the adoption of LECS for early gastric carcinoma will be very important in dealing with cases involving intra-mucosal carcinoma that would be difficult to treat by ESD due to ulcer scars or location, and in cases of submucosal carcinoma without lymph node metastasis.

## Conclusion

LECS for the stomach is an efficient, safe procedure that can preserve function. LECS for intra-mucosal gastric carcinoma is an efficient procedure, but strict observation is necessary because of the possibility of peritoneal dissemination. Results suggest that LECS is likely to be effective for cases involving intra-mucosal gastric carcinoma that are difficult to treat by ESD due to ulcer scars.
